# Arginase-1 and Treg Profile Appear to Modulate Inflammatory Process in Patients with Chronic Gastritis: *IL-33* May Be the Alarm Cytokine in *H. pylori*-Positive Patients

**DOI:** 10.1155/2019/2536781

**Published:** 2019-06-20

**Authors:** Emerson Abdulmassih Wood da Silva, Natalia Maria Jacom Wood da Silva, Rafael Rocha Rodrigues, Sheila Jorge Adad, Sanívia Aparecida de Lima Pereira, Betânia Maria Ribeiro, Mônica Sawan Mendonça, Fernanda R. Helmo, Virmondes Rodrigues, Denise Bertulucci Rocha Rodrigues

**Affiliations:** ^1^Laboratory of Biopathology and Molecular Biology, University of Uberaba (UNIUBE), Uberaba, MG, Brazil; ^2^Federal University of Triângulo Mineiro (UFTM), Uberaba, MG, Brazil; ^3^INCT-Neuroimmunomodulation, Brazil

## Abstract

*Helicobacter pylori* (*H. pylori*) is a highly prevalent bacterium in our environment, directly involved in various upper digestive tract diseases, such as gastritis, peptic ulcer, and gastric cancer. Several molecules activating the immune system have been reported to be involved in containing *H. pylori* infection. This study is aimed at analyzing the mRNA expression of the cytokines *IFN-γ*, *IL-17*, *IL-10*, *TGF-β*, *IL-6*, *IL-22*, *IL-23*, and *IL-33*; transcription factors *T-bet*, *RORC*, and *FOXP3*; enzymes *ARG1*, *ARG2*, and *NOS2*; and neuropeptides *VIP* and *TAC* and their respective receptors *VIPR1* and *TACR1* in the stomach lining of patients with severe digestive disorders. One hundred and twenty six patients have been evaluated, presenting with symptoms in the upper digestive tract, with the clinical indication for an Upper Digestive Endoscopy exam. Two fragments of the mucosa of the gastric body and antrum have been collected for anatomopathological examination and to analyze the expression of enzymes, cytokines, and transcription factors using qPCR. Expression of the *ARG1* gene was seen as significantly higher in the group of patients with chronic inactive gastritis than in the control group. Expression of the *TGF-β* gene and its *FOXP3* transcription factor was significantly higher in the group of chronic inactive gastritis patients than in the control. Expression of *IFN-γ*, *IL-17*, *IL-10*, and *TGF-β* and the transcription factors, *T-bet* and *RORC*, in the presence or absence of *H. pylori* showed no significant difference. However, the expression of *FOXP3* was significantly lower in *H. pylori*-positive patients than that in *H. pylori*-negative patients. ARG1 and Treg profile appeared to be modulating the inflammatory process, protecting patients from the tissue lesions with chronic inactive gastritis. Furthermore, we suggest that *IL-33* may be a crucial mediator of the immune response against an infection, after gastric mucosal damage.

## 1. Introduction

*Helicobacter pylori (H. pylori)* is a gram-negative spiral bacterium that colonizes the gastroduodenal mucosa of humans [1]. *H. pylori* infection represents a key factor in the etiology of various gastrointestinal diseases, from chronic active gastritis without clinical symptoms, to peptic ulceration, gastric adenocarcinoma, and the lymphoma of lymphoid tissue associated with gastric mucosa [2]. Although half of the world's population is infected with this bacterium, 80% of such individuals do not manifest the disease symptoms [3]. Studies have showed that the interaction between *H. pylori* subtype and the host immune response profile contributes to the chronicity of the disease and progression to more severe gastric disorders, thus explaining the different clinical manifestations of the infection caused by this microorganism [4, 5]. In the stomach, *H. pylori* enters the gastric mucus layer, not invading the gastric tissue; thus, the contact between *H. pylori* and phagocytic cells probably occurs infrequently, unless there exist ruptures in the epithelial gastric barrier [6]. It is believed that the microenvironment can result in a spectrum of phenotypes and macrophage functions *in vivo*; furthermore, classical M1 and an alternative M2-activated macrophage phenotypes are at the extremes of that spectrum [7]. Depending on the type of pathogens (pathogen-associated molecular patterns (PAMPs) or damage-associated molecular patterns (DAMPs)) and an environment rich in IFN-*γ*, the infection or tissue injury promotes the classic activation of highly proinflammatory type 1 M1 macrophages, expressing high levels of nitric oxide synthase (*NOS2*), interleukin- (IL-) 1*β*, and tumor necrosis factor (TNF), aimed at the destruction of the pathogens [8]. Nitric oxide (NO) can kill the bacteria *in vitro*; yet, the survival of *H. pylori* appears to be dependent on the amount of NO produced, which further depends on the availability of L-arginine as its substrate [9]. Alternately, the activation of type 2 macrophage (M2), which produce arginase, favors the healing and repair of lesions, with the secretion of high levels of *IL-10* and *TGF-β* [10, 11]. The balance of the expression of *NOS2* and arginase defines iNOS-dependent microbicidal capacity in an inflammatory environment; the deletion of arginase 2 (*ARG2*) in an experimental model results in increased gastritis and decreased bacterial load during *H. pylori* infection [12].

The interaction between *H. pylori* strains and the host immune response profile contributes to the chronicity of the disease and/or the progression to the more severe gastric disorders; this may explain the different clinical manifestations of the infection caused by this microorganism [4]. Although it is known that *H. pylori* infection triggers a predominantly Th1-like response [3, 4], the role of Th1 cells in *H. pylori* infection is not fully understood [13]. In addition to the Th1 profile, the Th17 profile may be associated with the regulation of stomach colonization by *H. pylori* [14]. Since *IL-23* is an important cytokine in the Th17 profile maintenance, the expression of *IL-23* after *H. pylori* infection appears to increase the recruitment of neutrophils at the site of infection [15]. The Treg profile associated with *TGF-β* production is described in the pathogenesis of the inflammatory process of the gastric mucosa [16]. This cytokine increases the production of Treg cells through the positive regulation of the *FOXP3* transcription factor, inhibiting the activation and proliferation of antigen-specific T cells and exerting an anti-inflammatory effect [16]. In the normal gastric mucosa, *TGF-β* plays an important role in the mucosal homeostasis maintenance under physiological conditions [17]. Nevertheless, the increase in the *TGF-β* expression in the gastric mucosa is important to control the inflammation induced by the bacterium. However, it is believed that *H. pylori* may secrete some soluble proteins that induce *TGF-β* production in the gastric epithelial cells and monocytes, favoring the colonization of host cells, thus contributing to the pathogenesis of the infection [18]. Considerably suppressed levels of *TGF-β1* have been observed in individuals exposed to *H. pylori*; this decrease was associated with *H. pylori* infection in patients with peptic ulcers [19]. Generally, TGF-*β* is a multifunctional cytokine that plays important roles in gastric inflammation through various regulatory mechanisms. Furthermore, gastric mucosa presents capsaicin-sensitive sensory nerve (CSSN)—its activation can be involved in symptoms and in the modulation of the inflammatory process. Neuropeptides are involved in the signaling of CSSN and may play an important role in the modulation of the immune response. Among these, vasoactive intestinal polypeptide (VIP) and substance P (SP) have anti- and proinflammatory effects, respectively [20].

Another cytokine studied in response to *H. pylori* infection is *IL-33*; however, its mechanism of action is not yet fully understood [21]. It has been indicated as an alarming cytokine against *H. pylori* infection. The expression of *IL-33* mRNA was shown to be significantly greater in biopsies of patients with *H. pylori* infection, as compared to the uninfected patients. Additionally, *IL-33* mRNA expression levels were significantly lower in patients with chronic gastritis, as compared to those with active gastritis [22].

In the present study, the expression of the cytokine genes *IFN-γ*, *IL-17*, *IL-10*, *TGF-β*, *IL-6*, *IL-22*, *IL-23*, and *IL-33*; transcription factors *T-bet*, *RORC*, and *FOXP3*; enzymes *ARG1*, *ARG2*, and *NOS2*; and neuropeptides *VIP* and *TAC* and their respective receptors *VIPR1* and *TACR1* in the stomach fragments of patients with upper digestive tract symptoms was compared in the presence or absence of *H. pylori*.

## 2. Materials and Methods

### 2.1. Sample Characterization

126 patients with symptoms of the upper digestive tract disorder and with the clinical indication of the Upper Digestive Endoscopy (UDE) examination were selected. Patients were randomized; those using Proton Pump Inhibitors (PPIs), histamine blockers (H2 blockers), antibiotics, or corticosteroids were instructed to discontinue the medication at least two weeks prior to the UDE exam. The UDE exams were performed at the Federal University of the Triângulo Mineiro (UFTM). Biopsies of the esophageal distal mucosa, gastric body, and antrum were collected from all patients during examination, according to present standardization in the literature and procedures established by the Brazilian Society of Digestive Endoscopy (SOBED). Fragments from the biopsies of the distal third of the esophagus, gastric body, and antrum were fixed in 4% buffered formalin, wherein 5 *μ*m thick histological sections were cut; the slides were stained with hematoxylin-eosin (HE) for routine histopathological study or with the silver-based Warthin-Starry technique for *H. pylori* detection. The histological findings of the gastric mucosa were interpreted according to the Sidney classification, with the modifications/graduation proposed by the Houston meeting [23]. An additional fragment of the gastric body mucosa was collected and placed in RNA*later*® (Life Technologies Corporation®, USA) for RNA extraction.

The patients were grouped according to the anatomopathological study (APS) of the gastric body regions; 44 patients had active chronic gastritis, 20 had chronic inactive gastritis, and 32 were without gastritis but with some inflammatory process in the antrum. The control group of 30 did not present any type of inflammatory process in any analyzed region of the stomach (body and antrum). This project was approved by the Research Ethics Committee (REC) of the University of Uberaba (UNIUBE), report number 350.874.

### 2.2. mRNA Extraction and Reverse Transcription

The gastric body fragments were thawed, transferred to sterile Eppendorf vials, and crushed manually with sterile metal pistils to obtain mRNA using the total RNA Isolation System SV kit (Promega, USA), according to the manufacturer's guidelines. Each sample was macerated, resuspended in 175 *μ*L Lysis Buffer, and homogenized in the vortex. After the addition of 350 *μ*L Dilution Buffer, the samples were placed in a water bath at 70°C for 3 min, and then centrifuged at 13000 ×*g* for 3 min. Further steps consisted of the addition of different solutions, followed by centrifugations at 13000 ×*g* for 1 minute, in the following order: addition of 200 *μ*L of 95% ethanol, centrifugation; 600 *μ*L RNA wash solution, centrifugation; 50 *μ*L of DNase Incubation Mix (consisting of 40 *μ*L Yellow Core Buffer, 5 *μ*L MnCl_2_, and 5 *μ*L DNase I per sample), incubation at room temperature for 15 min; addition of 200 *μ*L of DNase Stop Solution, centrifugation; 600 *μ*L RNA wash solution, centrifugation; and 250 *μ*L RNA wash solution, centrifugation. After the last phase, the basket containing the RNA was transferred to an eluate tube and 30 *μ*L of nuclease-free water was added; thereafter, centrifugation was followed at 13000 ×*g* for 1 minute to obtain the mRNA. In the end, the mRNA was quantified in a NanoDrop® 2000 spectrophotometer (Thermo Scientific) and stored in a freezer at -80°C until reverse transcription. For the preparation of complementary DNA (cDNA), the high-capacity cDNA reverse transcription kit (Applied Biosystems, USA) was used, according to the manufacturer's instructions. First, the RT Master Mix was prepared for each sample—its components are as follows: 2 *μ*L RT buffer, 0.8 *μ*L dNTP Mix, 2 *μ*L random primers, 1 *μ*L MultiScribe reverse transcriptase, and 4.2 *μ*L nuclease-free water, totaling 10.0 *μ*L per reaction. Then, 10 *μ*L of the RT Master Mix and 10 *μ*L of the mRNA were mixed and placed in a thermocycler in the following conditions: 25°C for 10 min, 37°C for 120 min, and 85°C for 5 min. In the end, the cDNA was quantified using a NanoDrop® ND1000 spectrophotometer and stored at -20°C until further use.

### 2.3. qPCR

The quantitative expression of the *ARG1*, *ARG2*, *NOS2*, *VIP*, *VIPR1*, *TAC*, *TACR1*, *IFN-γ*, *IL-17*, *IL-10*, *TGF-β*, *IL-6*, *IL-22*, *IL-23*, *IL-33*, *T-bet*, *RORC*, and *FOXP3* was analyzed by qPCR in the StepOnePlus™ Real-Time PCR Systems (Applied Biosystems, Foster, CA, USA) using the threshold cycle comparison method.

All experiments were mounted on 96-well plates of MicroAmp® Fast 96-Well Reaction Plate (Applied Biosystems, Foster, USA), and probes from the TaqMan system (Applied Biosystems, USA) were used, following the manufacturer's guidelines. The reaction used the following components: 5.0 *μ*L of TaqMan® Universal PCR Master Mix (reference 4304437, Applied Biosystems, USA), 0.5 *μ*L of TaqMan® Gene Expression Assay (probes), and 4.5 *μ*L of cDNA, per sample, totaling 10 *μ*L in each well. After assembling the plates, they were sealed with an adhesive (MicroAmp^tm^ Optical Adhesive Film—Thermo Scientific), centrifuged, and placed in the previously programmed thermal cycler. The amplification reaction comprised 2 min at 50°C, 10 min at 95°C for polymerase activation, and 40 cycles of 95°C for 15 s and of 60°C for 1 min, ideal temperatures for denaturation and annealing, respectively, of cDNA strands. For each cDNA sample, an amplification curve of each elected target and an internal gene calibrator (*β*-actin) was obtained. The results were analyzed based on the value of the CT (cycle threshold)—cycle threshold comparative; this is the point corresponding to the number of cycles where the amplification reaches a given threshold. The arithmetic form to obtain the relative quantification of DNA was QR = 2^−ΔΔCt^.

### 2.4. Statistical Analysis

Data was analyzed using the StatView® software (Abacus concept, USA) and IBM SPSS Statistics® (IBM Corporation®) (Version 23). The continuous variables were submitted to normality and variance (Kolmogorov-Smirnov) tests. Nonparametric tests were applied to analyze relative levels of mRNA. The variables presented a nonnormal distribution. They were analyzed by the nonparametric Mann-Whitney tests for comparison between two groups or Kruskal-Wallis followed by the Dunn posttest for comparison between three or more groups. Data was expressed as median, with minimum and maximum values and percentiles. Results were considered statistically significant when *p* < 0.05.

## 3. Results

### 3.1. Patient Assessment

We evaluated 126 biopsy samples of the gastric body of patients with upper digestive tract complaints. The mean age was 44.90 (±15.5) years, with 84 females age 44.9 (±15.9) years and 42 males age 44.8 (±14.9) years.

*H. pylori* was present in the gastric antrum of 42 (33.3%) and in the gastric body of 43 (34.1%) of the 126 patients studied, as determined by APS.

Among the 126 participants of the clinical questionnaire (Table 1), no significant difference was found in relation to the clinical data with the presence of *H. pylori* in the gastric antrum diagnosed by APS.

Based on anamnesis alone and in clinical situations such as epigastric pain, heartburn, smoking, alcoholism, obesity, and diabetes among others, a previous diagnosis of the *H. pylori* infection was not obtained. Epigastric pain was the most frequent symptom, affecting 105 (83.3%) patients, followed by heartburn, manifested in 86 (68.3%) patients (*p* > 0.05) (Table 1).

On comparing male and female patients harboring *H. pylori* in the gastric antrum, a higher prevalence of the bacterium in females was observed, although not statistically significant. Of the 84 (66.7%) female patients, 28 (22.2%) were positive for the presence of bacteria in the gastric antrum, while of the 42 (33.3%) males, 14 (11.1%) were positive to the gastric antrum (Table 1).

There existed no statistically significant difference when we compared the patients who had undergone previous *gastroesophageal reflux disease* (GERD) treatment. Of the 126 participants, 48 (38.1%) had been treated for GERD; among them, 16 (12.7%) had the bacteria present in the gastric antrum, while 32 (25.4%) were not positive for *H. pylori*.

For analysis of gene expression, samples were grouped according to the inflammatory process in the control, mean age was 43.50 (±15.7) years (19 females and 11 males); no gastritis, mean age was 44.70 (±15.1) years (21 females and 11 males); active gastritis, mean age was 44.0 (±15.0) years (29 females and 15 males); and chronic inactive gastritis, mean age was 49.0 (±17.3) years (15 females and 5 males), groups.

### 3.2. Analysis of Relative Gene Expression

**Figure 1:**
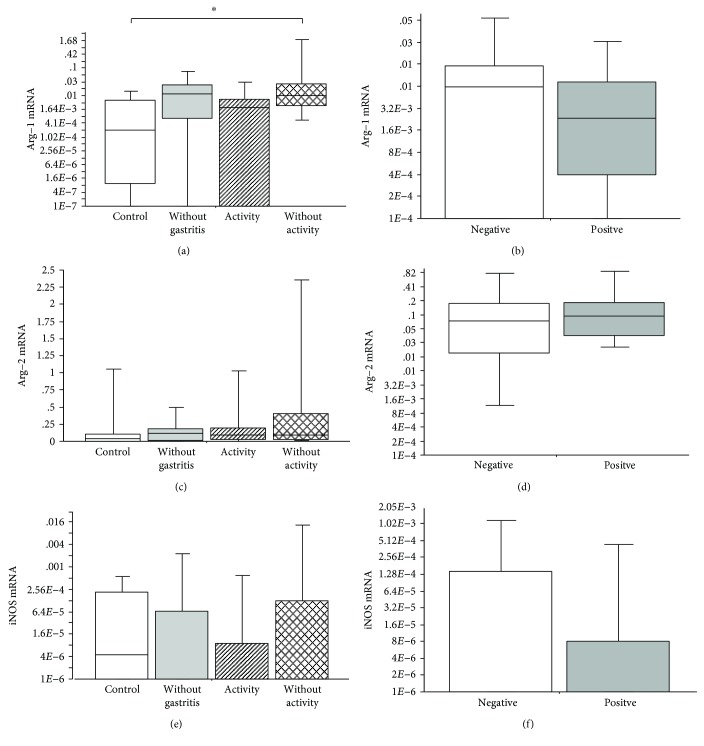
(a) Number of relative mRNA copies of *ARG1* present in biopsies of patients with chronic active gastritis, chronic inactive gastritis, no gastritis, or the control group (Kruskal-Wallis; *p* = 0.02); (b) number of relative mRNA copies of *ARG1* present in biopsies of patients with or without *H. pylori* (Mann-Whitney; *p* > 0.05); (c) number of relative mRNA copies of *ARG2* present in biopsies of patients with chronic active gastritis, chronic inactive gastritis, no gastritis, or the control group (Kruskal-Wallis; *p* > 0.05). (d) Number of relative mRNA copies of *ARG2* present in biopsies of patients with or without *H. pylori* (Mann-Whitney; *p* > 0.05); (e) number of relative mRNA copies of *iNOS* present in biopsies of patients with chronic active gastritis, chronic inactive gastritis, no gastritis, or the control group (Kruskal-Wallis; *p* > 0.05); (f) number of relative mRNA copies of *iNOS* present in biopsies of patients with or without *H. pylori* (Mann-Whitney; *p* > 0.05). The horizontal line represents the median, the bar percentile of 25%–75%, and the vertical line represents the percentile of 10%–90%. ^∗^ indicates significant *p* value.

The analysis of the gene expression of *ARG1*, *ARG2*, and *NOS2* was performed in the patients with chronic active gastritis, chronic inactive gastritis, no gastritis, or the control group. It was observed that the *ARG1* gene expression was significantly upregulated in the group of patients with chronic inactive gastritis, as compared to the control group (Kruskal-Wallis; *p* = 0.02) (Figure 1(a)). The other groups showed no significant difference (Kruskal-Wallis; *p* > 0.05) (Figures 1(c) and 1(e)). Additionally, these three genes were analyzed in the patient groups and classified by APS as *H. pylori*-positive or *H. pylori*-negative. No significant difference was observed on comparing the expression of *ARG1*, *ARG2*, or *NOS2* genes in the presence or absence of *H. pylori* (Mann-Whitney; *p* > 0.05) (Figures 1(b), 1(d), and 1(e)).

**Figure 2:**
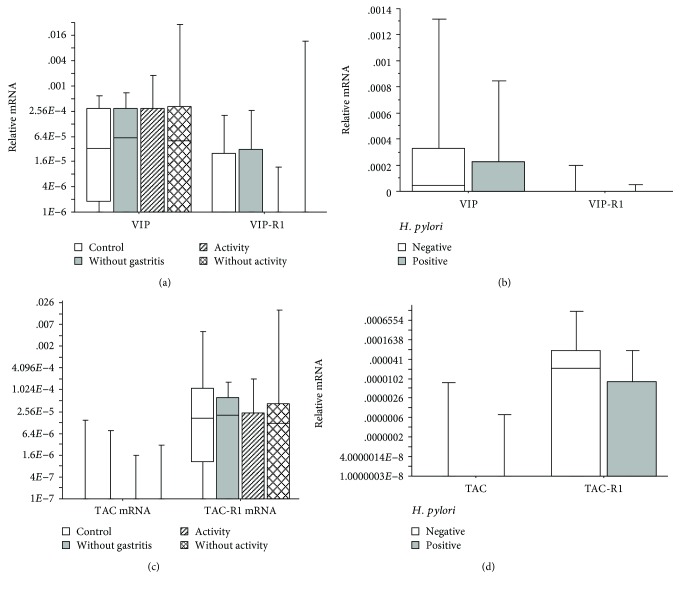
(a) Number of relative mRNA copies of *VIP* and *VIPR1* present in biopsies of patients with chronic active gastritis, chronic inactive gastritis, no gastritis, or the control group (Kruskal-Wallis; *p* > 0.05); (b) number of relative mRNA copies of *VIP* and *VIPAC* present in biopsies of patients with or without *H. pylori* (Mann-Whitney; *p* > 0.05); (c) number of relative mRNA copies of *TAC* and *TACR1* present in biopsies of patients with chronic active gastritis, chronic inactive gastritis, no gastritis, or the control group (Kruskal-Wallis; *p* > 0.05). (d) Number of relative mRNA copies of *TAC* and *TACR1* present in biopsies of patients with or without *H. pylori* (Mann-Whitney; *p* > 0.05). The horizontal line represents the median, the bar percentile of 25%–75%, and the vertical line represents the percentile of 10%–90%. ^∗^ indicates significant *p* value.

Furthermore, the expression of *VIP*, *VIPR1*, *TAC*, and *TACR1* was analyzed in patients with chronic active gastritis, chronic inactive gastritis, no gastritis, or the control group; no significant difference was observed among the groups (Kruskal-Wallis; *p* > 0.05) (Figures 2(a) and 2(c)). No significant difference was observed on comparing the expression of *VIP*, *VIPR1*, *TAC*, and *TACR1* genes in the presence or absence of *H. pylori* (Mann-Whitney; *p* > 0.05) (Figures 2(b) and 2(d)).

**Figure 3:**
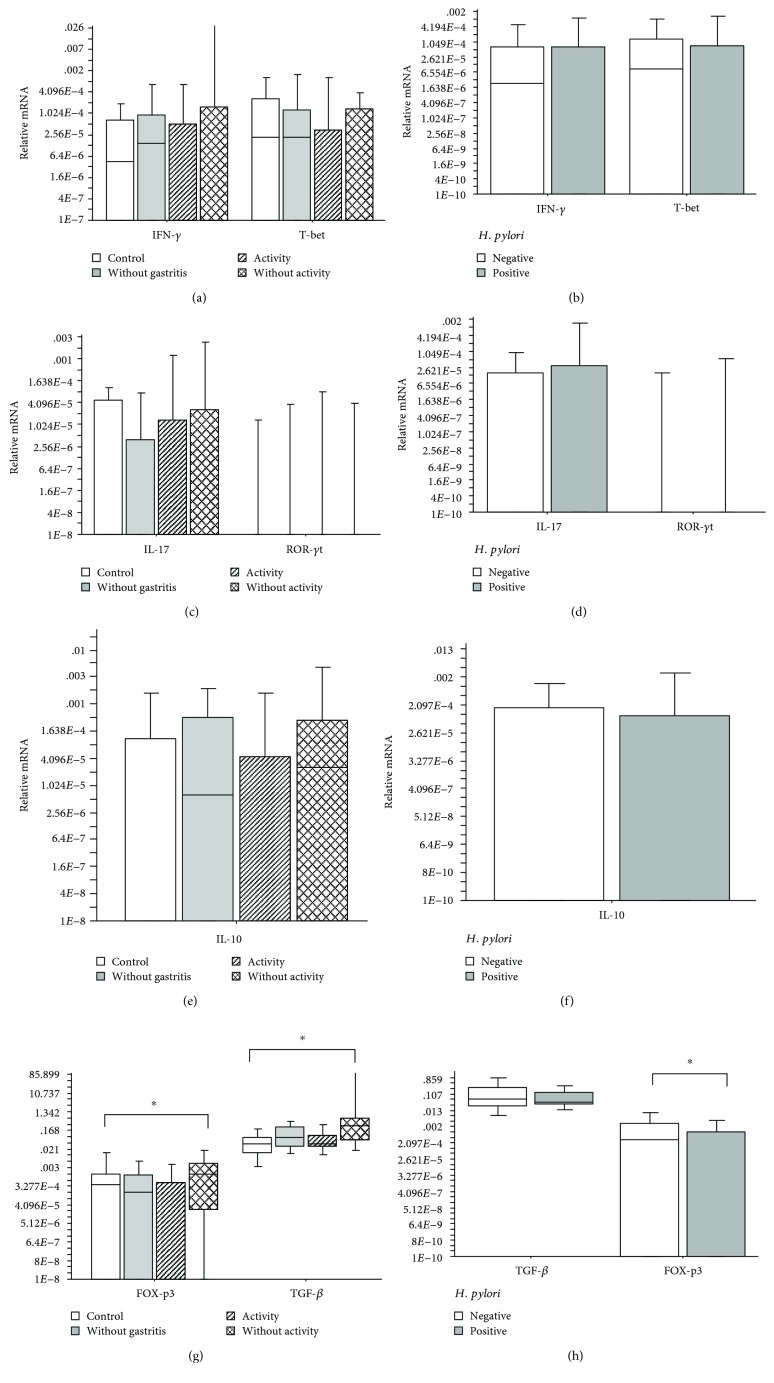
(a) Number of relative mRNA copies of *IFN-γ* and *T-bet* present in biopsies of patients with chronic active gastritis, chronic inactive gastritis, no gastritis, or the control group (Kruskal-Wallis; *p* = 0.02); (b) number of relative mRNA copies of *IFN-γ* and *T-bet* present in biopsies of patients with or without *H. pylori* (Mann-Whitney; *p* > 0.05); (c) number of relative mRNA copies of *IL-17* and *RORC* present in biopsies of patients with chronic active gastritis, chronic inactive gastritis, no gastritis, or the control group (Kruskal-Wallis; *p* > 0.05); (d) number of relative mRNA copies of *IL-17* and *RORC* present in biopsies of patients with or without *H. pylori* (Mann-Whitney; *p* > 0.05); (e) number of relative mRNA copies of *IL-10* present in biopsies of patients with chronic active gastritis, chronic inactive gastritis, no gastritis, or the control group (Kruskal-Wallis; *p* > 0.05); (f) number of relative mRNA copies of *IL-10* present in biopsies of patients with or without *H. pylori* (Mann-Whitney; *p* > 0.05); (g) number of relative mRNA copies of *FOXP3* and *TGF-β* present in biopsies of patients with chronic active gastritis, chronic inactive gastritis, no gastritis, or the control group (Kruskal-Wallis; *p* = 0.02; *p* = 0.03, respectively); (h) number of relative mRNA copies of *TGF-β* and *FOXP3* present in biopsies of patients with or without *H. pylori* (Mann-Whitney; *p* > 0.01). The horizontal line represents the median, the bar percentile of 25%–75%, and the vertical line represents the percentile of 10%–90%. ^∗^ indicates significant *p* value.

Similarly, while analyzing the expression of the *IFN-γ*, *IL-17*, and *IL-10* genes and the *T-bet* and *RORC* transcription factors, in patients with chronic active gastritis, chronic inactive gastritis, no gastritis, or the control group, no significant difference was observed (Kruskal-Wallis; *p* > 0.05). Nevertheless, the expression of the *TGF-β* gene and its *FOXP3* transcription factor was significantly higher in patients with chronic inactive gastritis, as compared to the control group (Kruskal-Wallis; *p* = 0.02 and *p* = 0.03, respectively) (Figure 3(g)). On comparing the expression of the *IFN-γ*, *IL-17*, *IL-10*, and *TGF-β* and the *T-bet* and *RORC* transcription factors in the presence or absence of *H. pylori*, no significant difference was observed (Mann-Whitney; *p* > 0.05) (Figures 3(b), 3(d), and 3(f)). However, the *FOXP3* expression was significantly lower in the *H. pylori*-positive patients, as compared to the *H. pylori*-negative patients (Mann-Whitney; *p* = 0.01) (Figure 3(h)).

**Figure 4:**
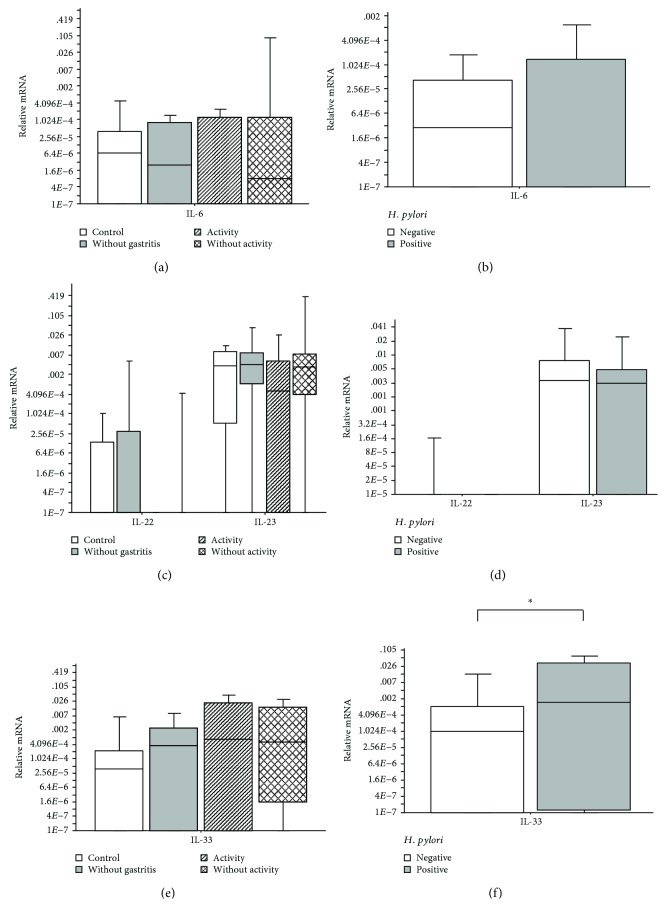
(a) Number of relative mRNA copies of *IL-6* present in biopsies of patients with chronic active gastritis, chronic inactive gastritis, no gastritis, or the control group (Kruskal-Wallis; *p* > 0.05); (b) number of relative mRNA copies of *IL-6* present in biopsies of patients with or without *H. pylori* (Mann-Whitney; *p* > 0.05); (c) number of relative mRNA copies of *IL-22* and *IL-23* present in biopsies of patients with chronic active gastritis, chronic inactive gastritis, no gastritis, or the control group (Kruskal-Wallis; *p* > 0.05); (d) number of relative mRNA copies of *IL-22* and *IL-23* present in biopsies of patients with or without *H. pylori* (Mann-Whitney; *p* > 0.05); (e) number of relative mRNA copies of *IL-33* present in biopsies of patients with chronic active gastritis, chronic inactive gastritis, no gastritis, or the control group (Kruskal-Wallis; *p* > 0.05); (f) number of relative mRNA copies of *IL-33* present in biopsies of patients with or without *H. pylori* (Mann-Whitney; *p* = 0.03). The horizontal line represents the median, the bar percentile of 25%–75%, and the vertical line represents the percentile of 10%–90%. ^∗^ indicates significant *p* value.

On analyzing the gene expression of *IL-6*, *IL-22*, *IL-23*, and *IL-33* in patients with chronic active gastritis, chronic inactive gastritis, no gastritis, or the control group, no significant difference was observed (Kruskal-Wallis; *p* > 0.05). However, the *IL-33* gene expression was significantly upregulated in the *H. pylori*-positive patients, as compared to the *H. pylori*-negative patients (Mann-Whitney; *p* = 0.03) (Figure 4(f)).

## 4. Discussion

The present study analyzed the mRNA expression of a set of mediators involved in inflammatory response modulation. The expression of genes encoding cytokines, chemokines, neuropeptides, and enzymes involved in the effector mechanisms and the regulators of inflammation was analyzed. The study evaluated gastric body biopsies obtained during the digestive endoscopy examination, performed in patients with dyspepsia. We observed a significant upregulation in the expression of the *ARG1* gene in the group of patients with chronic inactive gastritis, as compared to the control group. The *ARG1* enzyme, which catalyzes the hydrolysis of arginine to ornithine and urea, is produced in the liver under the stimulation of *IL-4*, *IL-10*, and *TGF-β*, as well as by immune response cells, such as activated neutrophils at infection sites. It can modulate the immune response and functions in the process of tissue regeneration [24]. In our study, *ARG1* association with a type of chronic inactive gastritis suggested that it may be involved in the regulation of inflammation and the repair processes arising in acute inflammatory lesions. In our study, no significant difference was found in the expression of the genes *ARG2* and *NOS2*, which also have L-arginine as the substrate. *ARG2* hydrolyzes L-arginine in the same way as *ARG1*, while *NOS2* produces nitric oxide, a potent microbicidal agent associated with the growth inhibition of microorganisms, causing tissue damage in the process. Thus, the association of *ARG1* with chronic inflammation may indicate an ongoing repair process [25]. Conversely, some authors associate the presence of arginases, especially *ARG2*, with the escape mechanisms of *H. pylori*, by causing a low availability of L-arginine and thus inhibiting the NOS2-dependent microbicidal activity. Moreover, the expression of arginase by *H. pylori* itself was associated with its carcinogenic activity [26]. We believe that the patients with chronic inactive gastritis established a mechanism of protection against infection, and with an increase in *ARG1*, established a modulation process for regeneration.

The *TGF-β* and *FOXP3* transcription factor expression was significantly higher in the group of chronic inactive gastritis patients, as compared to the control group. In an *H. pylori* infection, when the Th1 profile response was predominant, there was an establishment of the effector mechanisms that reduced the number of bacteria; however, a greater epithelial damage could occur with the subsequent progression of gastritis to gastric atrophy. *H. pylori* has an important interface with *TGF-β*, since it can induce *TGF-β* production in a VacA-dependent manner [27]; furthermore, it has a potential modulating effect on its signaling, through the interaction between CagA and Smad3 [28]. The results presented here, in which a high mRNA expression of *TGF-β* is associated with chronic inactive gastritis, suggest that the cytokine is associated with mechanisms of inflammation control and regeneration processes.

Corroborating this hypothesis, we found a low mRNA expression of *FOXP3* in *H. pylori*-positive samples. *FOXP3* encodes a signaling molecule associated with Treg cells, the process of regulation of the immune response; generally, its expression is less in the presence of the bacterium, suggesting it can repress its production, reducing the pressure of immunoregulation and the establishment of effector mechanisms with the potential ability to control bacterial growth. It is important to consider that these same mechanisms have a potential effect of damaging the mucosa.

We found no significant difference in the gene expression of *IFN-γ*, *IL-17*, and *IL-10* and the *T-bet* and *RORC* transcription factors in the groups of patients with chronic active gastritis, chronic inactive gastritis, no gastritis, or the control group; the same was noted when we compared to the group of *H. pylori*-positive or *H. pylori* -negative patients. Alternately, some studies observed an upregulation of *IL-17* and downmodulation of Treg cells in peptic ulcer associated with *H. pylori* infection [29, 30]. The differences in the observations between these two studies may be due to the characteristics of the sample pooling process. Moreover, previous studies have showed an association with peptic ulcer, a complication not observed in our study. Hence, we believe that the Treg profile seems to be modulating the inflammatory process of patients with chronic inactive gastritis, protecting them from tissue lesions and worsening of the gastritis.

Furthermore, in the present work, the *IL-33* gene expression was significantly upregulated in *H. pylori*-positive patients, as compared to the *H. pylori*-negative patients.

We also analyzed the gene expression of *IL-6*, *IL-22*, *IL-23*, and *IL-33* in patients with chronic active gastritis, chronic inactive gastritis, no gastritis, or the control group and found no significant difference. The *IL-33* has been described as an alarming indicator of tissue damage. It is highly expressed in the gastric mucosa and appears to potently activate Th2 immunity and the regulatory cells of the immune system [31]. Studies suggest that it may play a key role during *H. pylori* infection. Other studies have observed an increase in the expression of *IL-33* in biopsies of patients infected with *H. pylori*, as well as in cases of active gastritis [22]. *IL-33* production during an infection can modulate Th1- and Th17-exacerbated inflammation, contributing to lesser tissue damage. Its ability to activate Th2 response and regulation also induces differentiation of the M2 macrophages. These participate in tissue repair processes and express arginase, associated with chronic inactive gastritis in this study. The data suggest that *IL-33*, in addition to acting as an alarming cytokine against *H. pylori* infection, is associated with the repair mechanisms, production of the anti-inflammatory mediators, and the possible differentiation of M2 macrophages, modulating the inflammatory processes and stimulating repair in the injured tissues. Thus, *IL-33* may be a crucial mediator of the immune response in infection, after damage to the mucosal epithelial tissues.

## 5. Conclusion

Thus, ARG1 and Treg profile appear to be modulating the inflammatory process, protecting chronic inactive gastritis patients from tissue lesions. Furthermore, we suggest *IL-33* may be a crucial mediator of the immune response in infection, after gastric mucosal damage.

## Figures and Tables

**Table 1:** Comparison of clinical data with the presence of *H. pylori* studied in the gastric antrum through the PAD (*N* = 126).

Clinical data	Number	*H. pylori +*	*H. pylori* -
Male	42 (33.3%)	14 (11.1%)	28 (22.2%)
Women	84 (66.7%)	28 (22.2%)	56 (44.4%)
Epigastric pain	105 (83.3%)	35 (27.8%)	70 (55.6%)
Nausea	56 (44.4%)	21 (16.7%)	35 (27.8%)
Pirose	86 (68.3%)	27 (21.4%)	59 (46.8%)
Dysphagia	30 (23.8%)	15 (11.9%)	15 (11.9%)
Regurgitation	42 (33.3%)	17 (13.5%)	25 (19.9%)
Smoking	22 (17.5%)	6 (4.8%)	16 (12.7%)
Ethicism	50 (39.7%)	20 (15.9%)	30 (23.8%)
Obesity	27 (21.4%)	13 (10.3%)	14 (11.1%)
Diabetes	12 (9.5%)	5 (4.0%)	7 (5.6%)
Previous treatment GERD	48 (38.1%)	16 (12.7%)	32 (25.4%)
Previous treatment of gastritis	37 (29.4%)	8 (6.3%)	29 (23.0%)
Previous treatment *H. pylori*	19 (15.1%)	3 (2.4%)	16 (12.7%)

*p* < 0.05.

## Data Availability

The data used to support the findings of this study are available from the corresponding author upon request.

## References

[1] Warren J. R., Marshall B. (1983). Unidentified curved bacilli on gastric epithelium in active chronic gastritis. *The Lancet*.

[2] Kusters J. G., van Vliet A. H. M., Kuipers E. J. (2006). Pathogenesis of *Helicobacter pylori* infection. *Clinical Microbiology Reviews*.

[3] Suerbaum S., Michetti P. (2002). *Helicobacter pylori* infection. *The New England Journal of Medicine*.

[4] Vinagre R. M. D. F., Vinagre I. D. F., Vilar-e-Silva A., Fecury A. A., Martins L. C. (2018). *Helicobacter pylori* infection and immune profile of patients with different gastroduodenal diseases. *Arquivos de Gastroenterologia*.

[5] Estevam R. B., Wood da Silva N. M. J., Wood da Silva (2017). Modulation of galectin-3 and galectin 9 in gastric mucosa of patients with chronic gastritis and positive *Helicobacter pylori* infection. *Pathology - Research and Practice*.

[6] Algood H. M. S., Cover T. L. (2006). *Helicobacter pylori* persistence: an overview of interactions between *H. pylori* and host immune defenses. *Clinical Microbiology Reviews*.

[7] Xue J., Schmidt S. V., Sander J. (2014). Transcriptome-based network analysis reveals a spectrum model of human macrophage activation. *Immunity*.

[8] Martinez F. O., Gordon S. (2014). The M1 and M2 paradigm of macrophage activation: time for reassessment. *F1000Prime Reports*.

[9] Chaturvedi R., Asim M., Lewis N. D. (2007). L-arginine availability regulates inducible nitric oxide synthase-dependent host defense against *Helicobacter pylori*. *Infection and Immunity*.

[10] Benoit M., Desnues B., Mege J. L. (2008). Macrophage polarization in bacterial infections. *The Journal of Immunology*.

[11] Fleming B. D., Mosser D. M. (2011). Regulatory macrophages: setting the threshold for therapy. *European Journal of Immunology*.

[12] Hardbower D. M., Asim M., Murray-Stewart T. (2016). Arginase 2 deletion leads to enhanced M1 macrophage activation and upregulated polyamine metabolism in response to *Helicobacter pylori* infection. *Amino Acids*.

[13] Bagheri N., Salimzadeh L., Shirzad H. (2018). The role of T helper 1-cell response in Helicobacter pylori-infection. *Microbial Pathogenesis*.

[14] Bhuiyan T. R., Islam M. M. T., Uddin T. (2014). Th1 and Th17 responses to *Helicobacter pylori* in Bangladeshi infants, children and adults. *PLoS One*.

[15] Kabir S. (2011). The role of interleukin-17 in the *Helicobacter pylori* induced infection and immunity. *Helicobacter*.

[16] Curotto de Lafaille M. A., Lafaille J. J. (2009). Natural and adaptive foxp3^+^ regulatory T cells: more of the same or a division of labor?. *Immunity*.

[17] Hawinkels L. J. A. C., Verspaget H. W., van Duijn W. (2007). Tissue level, activation and cellular localisation of TGF-*β*1 and association with survival in gastric cancer patients. *British Journal of Cancer*.

[18] Wu M.-S., Lin J.-T., Hsu P.-N. (2007). Preferential induction of transforming growth factor—*β* production in gastric epithelial cells and monocytes by *Helicobacter pylori* soluble proteins. *The Journal of Infectious Diseases*.

[19] Jo Y., Han S. U., Kim Y. J. (2010). Suppressed gastric mucosal TGF-*β*1 increases susceptibility to *H. pylori*-induced gastric inflammation and ulceration: a stupid host defense response. *Gut and Liver*.

[20] Erin N., Türker S., Elpek O., Yıldırım B. (2012). Differential changes in substance P, VIP as well as neprilysin levels in patients with gastritis or ulcer. *Peptides*.

[21] Tran L. S., Tran D., de Paoli A. (2018). NOD1 is required for *Helicobacter pylori* induction of IL-33 responses in gastric epithelial cells. *Cellular Microbiology*.

[22] Shahi H., Reiisi S., Bahreini R., Bagheri N., Salimzadeh L., Shirzad H. (2015). Association between *Helicobacter pylori* cagA, babA2 virulence factors and gastric mucosal interleukin-33 mRNA expression and clinical outcomes in dyspeptic patients. *International Journal of Molecular and Cellular Medicine*.

[23] Dixon M. F., Genta R. M., Yardley J. H., Correa P. (1996). Classification and grading of gastritis. The updated Sydney system. International Workshop on the Histopathology of Gastritis, Houston 1994. *The American Journal of Surgical Pathology*.

[24] Jacobsen L. C., Theilgaard-Monch K., Christensen E. I., Borregaard N. (2007). Arginase 1 is expressed in myelocytes/metamyelocytes and localized in gelatinase granules of human neutrophils. *Blood*.

[25] da Cunha Colombo Tiveron L. R., da Silva I. R., da Silva M. V., Peixoto A. B., Rodrigues D. B. R., Rodrigues V. (2018). High in situ mRNA levels of IL-22, TFG-*β*, and ARG-1 in keloid scars. *Immunobiology*.

[26] Shi Y. Y., Chen M., Zhang Y. X., Zhang J., Ding S. G. (2014). Expression of three essential antioxidants of *Helicobacter pylori* in clinical isolates. *Journal of Zhejiang University Science B*.

[27] Rahimian G., Sanei M. H., Shirzad H. (2014). Virulence factors of *Helicobacter pylori* vacA increase markedly gastric mucosal TGF-*β*1 mRNA expression in gastritis patients. *Microbial Pathogenesis*.

[28] Nguyen T. T., Kim S. J., Park J. M., Hahm K. B., Lee H. J. (2015). Repressed TGF-*β* signaling through CagA-Smad3 interaction as pathogenic mechanisms of *Helicobacter pylori*-associated gastritis. *Journal of Clinical Biochemistry and Nutrition*.

[29] Bagheri N., Shirzad H., Elahi S. (2017). Downregulated regulatory T cell function is associated with increased peptic ulcer in *Helicobacter pylori*-infection. *Microbial Pathogenesis*.

[30] Bagheri N., Razavi A., Pourgheysari B. (2018). Up-regulated Th17 cell function is associated with increased peptic ulcer disease in *Helicobacter pylori*-infection. *Infection, Genetics and Evolution*.

[31] Schmitz J., Owyang A., Oldham E. (2005). IL-33, an interleukin-1-like cytokine that signals via the IL-1 receptor-related protein ST2 and induces T helper type 2-associated cytokines. *Immunity*.

